# Identification of circulating metabolites associated with chronic rhinosinusitis using Mendelian randomization analysis

**DOI:** 10.1016/j.bjorl.2025.101626

**Published:** 2025-04-25

**Authors:** Fan Jiang, Junhao Tu, Wenqi Luo, Yizhen Jia, Qing Luo, Jing Ye

**Affiliations:** a Nanchang University, Jiangxi Medical College, The First Affiliated Hospital, Department of Otorhinolaryngology, Head and Neck Surgery, Nanchang, Jiangxi Province, China; b Jiangxi Medicine Academy of Nutrition and Health Management, Nanchang, Jiangxi Province, China; c Nanchang University, Jiangxi Medical College, The First Affiliated Hospital, Department of Allergy, Nanchang, Jiangxi Province, China; d Nanchang University, Jiangxi Medical College, The First Affiliated Hospital, Institute of Otorhinolaryngology, Nanchang, Jiangxi Province, China

**Keywords:** Chronic rhinosinusitis, Mendelian randomization, Circulating metabolites

## Abstract

**Objective:**

This study aims to employ Mendelian Randomization (MR) analysis to investigate causal relationships between serum metabolites and CRS, identifying key pathogenic and protective factors and analyzing their mechanisms of action.

**Methods:**

Utilizing data from the Genome-Wide Association Studies (GWAS) database, employing two-sample MR analysis to investigate the potential causal relationship between 233 circulating metabolites with the occurrence of CRS. Inverse Variance Weighted (IVW) model, MR-Egger method, Weighted Median, and Weighted model were employed. Sensitivity analyses were conducted with Bonferroni correction. This research aims to elucidate the impact of metabolites on the development and progression of CRS, providing valuable insights into the underlying mechanisms.

**Results:**

Following MR analysis, two metabolites were significantly associated with CRS: Tyrosine (OR = 1.223; 95% CI 1.115–1.341; *p* = 1.96E-05) and Creatinine (OR = 1.208; 95% CI 1.103–1.322; *p* = 4.11E-05). These two key risk factors may be further studied for their pathogenesis and could be targeted for modulation in the treatment of CRS. However, there are several protective factors also worth exploring, among which the correlation is more significant: Ratio of conjugated linoleic acid to total fatty acids (OR = 0.809; 95% CI 0.708‒0.923; *p* = 1.73E-03), Albumin (OR = 0.787; 95% CI 0.670‒0.926; *p* = 3.76E-03),Conjugated linoleic acid (OR = 0.664; 95% CI 0.491‒0.898; *p* = 7.85E-03), Diacylglycerol (OR = 0.804; 95% CI 0.654‒0.989; *p* = 3.87E-02), Apolipoprotein A–I (OR = 0.915; 95% CI 0.845‒0.991; *p* = 2.89E-02).

**Conclusion:**

In our MR study, we discovered 28 circulating metabolites linked to CRS. Importantly, tyrosine and creatinine were identified as the most significant contributors to the pathogenesis of CRS, highlighting their potential as therapeutic targets. Additionally, several protective factors may offer new avenues for preventive strategies and therapeutic interventions. These findings underscore the clinical relevance of targeting these metabolites to modulate CRS progression and improve patient outcomes.

**Level of evidence:**

Level 2*.^1^

## Highlights

• Analyzed chronic rhinosinusitis and metabolites using Mendelian randomization.
• Tyrosine and creatinine are the main pathogenic factors.
• 24 circulating metabolites are protective factors.

## Introduction

Chronic Rhinosinusitis (CRS) is a common upper airway disease characterized by persistent locoregional mucosal inflammation.^2^ It significantly affects the quality of life of patients, with an incidence rate of approximately 6.9%‒27.1% in Europe and around 4.8%‒9.7% in China.^3^, ^4^ It incurs higher medical expenses and leads to decreased productivity and work efficiency.^5^ Therefore, understanding the pathogenesis of this disease plays a crucial role in its prevention.

The potential association of CRS with serum metabolites, such as the dysregulation of the arachidonic acid metabolic pathway in circulating metabolites, leading to the development of CRS with Nasal Polyps (CRSwNP).^6^ Circulating metabolites play a crucial role in the infiltration of inflammatory cells within the human body, directly influencing the development of various inflammatory diseases. Circulating ketone Body β-Hydroxybutyrate (βOHB) reduces the activation of pro-inflammatory macrophages.^7^ M2 macrophages Infiltration may aggravate epithelial remodeling of the nasal mucosa, accelerating CRS progression.^8^ Therefore, circulating metabolites may impact the local immune response in CRS by affecting immune cell function and systemic metabolism, particularly mitochondrial processes like glycolysis. Elevated glucose levels in nasal secretions of CRS patients can boost glycolysis in epithelial cells, increasing their pro-inflammatory activities.^9^, ^10^ Consequently, dysregulation of circulating metabolites may accelerate CRS progression by affecting glycolysis. Specifically, circulating metabolites activate a series of signaling pathways that promote the migration and infiltration of inflammatory cells. It can be observed in numerous diseases, such as atherosclerosis and arthritis. For instance, in atherosclerosis, cholesterol and fatty acids promote the migration of monocytes to the arterial wall, forming plaques and triggering inflammatory responses.^11^ The key circulating metabolites identified in our MR analysis may influence the inflammatory response in CRS through distinct mechanisms. Tyrosine can undergo various post-translational modifications. When there is an excess of Reactive Oxygen Species (ROS) in tissues, it is susceptible to oxidative and nitrative modifications, which have been observed in conditions such as inflammation and rheumatoid arthritis.^12^, ^13^ Therefore, tyrosine may influence the progression of inflammatory mechanisms through post-translational modifications. Protein post-translational modifications are often influenced by various enzymes. If specific translational types contributing to the exacerbation of CRS can be identified, targeting the active sites of these enzymes may reduce the adverse effects of post-translational modifications. The level of creatinine is associated with the T-cell Kinase (ITK) signaling pathway, which may contribute to immune dysregulation and exacerbate type 2 inflammation in CRS by affecting the further release of inflammatory factors from T-cells.^14^ Local inhibition of the ITK signaling pathway may represent a novel target for modulating immune responses in CRS. The Conjugated Linoleic Acid (CLA) influences systemic immunity by modulating immature thymocytes and mature peripheral blood T-cells. Furthermore, the local immune response in CRS is also affected by systemic T-cells.^15^ This suggests that CLA may play a crucial role in alleviating Th2-type immune responses. Local inhibition of the ITK signaling pathway may represent a novel target for modulating immune responses in CRS. Therefore, circulating metabolites may affect the levels of inflammatory cells and cytokines in CRS. However, the current impact on CRS remains unknown.

The Mendelian Randomization (MR) method offers unique advantages in epidemiological research. Compared to traditional Randomized Controlled Trials (RCTs) and observational studies, MR uses genetic variations as Instrumental Variables (IVs), effectively controlling for confounding factors and reverse causality, allowing for accurate assessment of causal relationships between exposure factors and diseases.^16^ MR is more cost-effective than RCTs, making it suitable for studying outcomes where RCTs are unethical or impractical to implement. This positions MR as an essential research tool that can yield reliable conclusions with limited resources.^17^ Currently, numerous studies have used MR to explore the relationships between CRS and various risk factors. One study evaluated the causal association between CRS and factors like smoking and obesity.^18^ Another study employing MR found a significant association between 16 types of microbiotas and the occurrence of CRS.^19^ The employment of MR enhances our understanding of the etiology of CRS and offers new insights for its prevention and treatment. Additionally, MR has been used to investigate the relationship between metabolic cycles and other diseases. Chen et al. used MR analysis in a multi-ethnic population to link circulating metabolites and urine metabolites to the development of allergic diseases such as allergic rhinitis and Asthma.^20^

This study aims to identify circulating metabolites associated with CRS risk by analyzing 233 metabolites. The goal is to uncover biomarkers for early detection and develop targeted therapies, ultimately improving patient outcomes and quality of life.

## Methods

### Study design

**Fig. 1:**
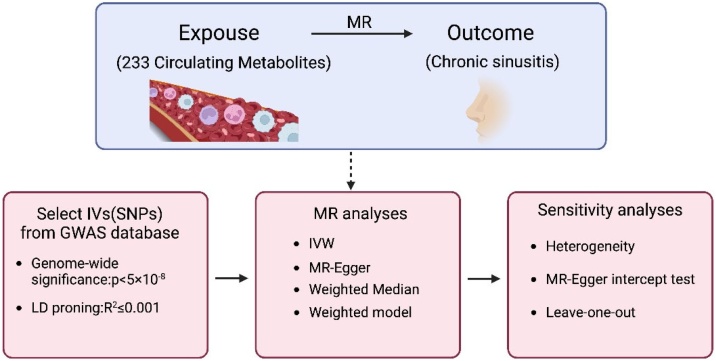
Study design.

To explore the potential relationship between CRS onset and circulating metabolites, we retrieved relevant CRS data from FinnGen database, which includes genetic and health information from over 500,000 individuals in Finland, focusing on those diagnosed with CRS.^21^ Conducting MR analysis comparing circulating metabolite levels in the GWAS database, encompassing diverse populations relevant to metabolic traits.^22^ MR uses genetic variation to test causal hypotheses in non-experimental data, like random allocation of treatment in a RCT, reducing confounding risks. Using Single Nucleotide Polymorphism (SNP) as IVs in the study. To ensure a strong association between SNPs and exposure factors with minimal confounding, we included SNPs that met the genome-wide significance threshold (*p* < 5 ×10^−8^) and performed Linkage Disequilibrium (LD) clumping with a 10,000 kb reference distance and *r*^2^ < 0.001 to maintain independence. SNPs not in Hardy-Weinberg equilibrium (*p* < 1 ×10^−6^) and those associated with potential confounders were excluded.^23^, ^24^ We prioritized SNPs in coding or regulatory regions, all genetic variants used as instrumental IVs were derived from populations consistent with the exposure and outcome datasets. This approach reduces reverse causation bias due to the immutability of genetic variations. Here, we first conducted MR analysis on 233 metabolites and CRS, followed by sensitivity analysis and reverse MR analysis (Fig. 1).

### Sources of data on circulating metabolites and CRS

Circulating metabolites was quantified by Minna et al.’s study.^25^ Employing nuclear magnetic resonance spectroscopy on a cohort of 136,016 participants, the study examined a comprehensive panel of 233 metabolites. This panel encompassed 213 lipids, lipoprotein parameters, and fatty acids, along with 20 non-lipid metabolites such as amino acids, ketone bodies, components of glycolysis/gluconeogenesis, markers of fluid balance, and metabolites associated with inflammation. Compared to the previous GWAS data, the sample size has increased significantly, including participants from 33 cohorts. The populations involved are more diverse, including Asians, Europeans, and individuals of African descent. The genetic data of CRS is sourced from the FinnGen database (tenth round), comprising 17,987 cases and 308,457 controls in the cohort. The project has collected and integrated genomic data and detailed health records of over 500,000 Finns, covering a wide range of diseases.

### Two-sample Mendelian randomization

In our investigation using two-sample MR analysis, we delved into the causal connection between 233 circulating metabolites and CRS. Adhering to three core principles: (1) Associativity, which posits a robust link between genetic variations and exposure factors; (2) Independence, asserting genetic variations remain unaffected by confounding factors influencing exposure and CRS; (3) Exclusivity, stipulating that genetic variations solely influence CRS through exposure factors, excluding other pathways.

Employing four analytical approaches ‒ IVW, MR-Egger method, Weighted Median, and Weighted model. (1) The IVW method is a statistical approach used in MR that combines the estimates from multiple genetic variants to assess the causal effect of an exposure on an outcome. Assuming that all variants are IVs and there is no horizontal pleiotropy.^26^ However, its sensitivity to invalid instruments limits its applicability, necessitating additional statistical methods. (2) The weighted model in MR enhances the IVW method by incorporating additional weights to account for potential pleiotropy and biases. Providing flexibility in managing invalid instruments and improves the robustness of causal estimates. Consequently, this leads to more accurate and dependable findings in causal inference.^27^ (3) The MR-Egger method can detect violations of the standard instrumental variable assumption, providing sensitivity analysis for the results.^28^ Comprising three components: testing for directional pleiotropy, causal effect testing, and causal effect estimation. It evaluates genetic variations for directional pleiotrop, but it may be biased and inflate the type I error rate.^29^ (4). Weighted Median is a supplement to the MR-Egger regression method, which can combine data from multiple genetic variations into a single causal estimate. If at least 50% of the weight comes from valid Independent Variables (IV), then the weighted median provides a consistent estimate.^30^

### Sensitivity analysis

We applied Bonferroni correction to the data to reduce the risk of Type I error. A result is considered statistically significant when *p* < 0.05/233 = 2.00E-4. When 0.05 > *P* > 0.05/233 = 2.00E-4, the result is considered suggestive.

## Results

### Threshold of instrumental variables and SNPs

A total of 13,360 SNPs were extracted through original GWAS data with a significance level of *p* < 5.00E-8. Using *r*^2^ > 0.001 and 10,000 kb as a reference to exclude linkage disequilibrium, the remaining 12,341 SNPs were used for analysis (Table S1 in Supplementary material).

### Casual relationships of circulating metabolites and CRS

According to the MR analysis results, total 28 circulating metabolites are (positively/negatively) associated with the risk of CRS. After correction, two metabolites were found to be statistically significant: Tyrosine (OR = 1.223; 95% CI 1.115–1.341; *p* = 1.96E-05) and Creatinine (OR = 1.208; 95% CI 1.103–1.322; *p* = 4.11E-05), which are risk factors for CRS onset.

**Fig. 2:**
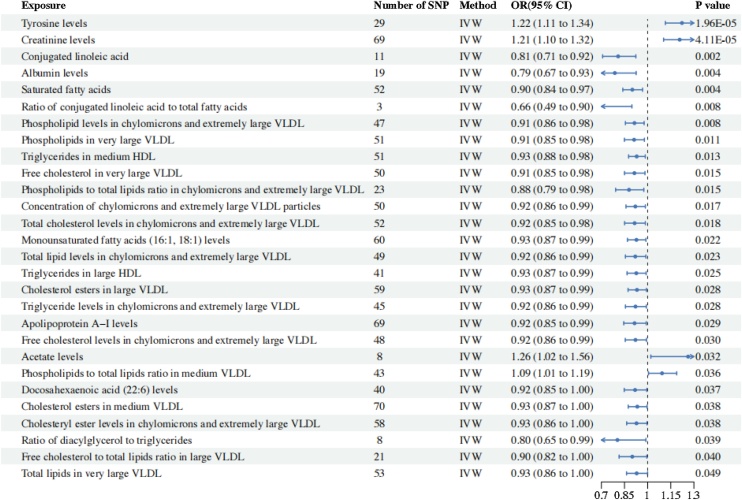
IVW analysis of 28 Circulating metabolites associated with CRS.

**Fig. 3:**
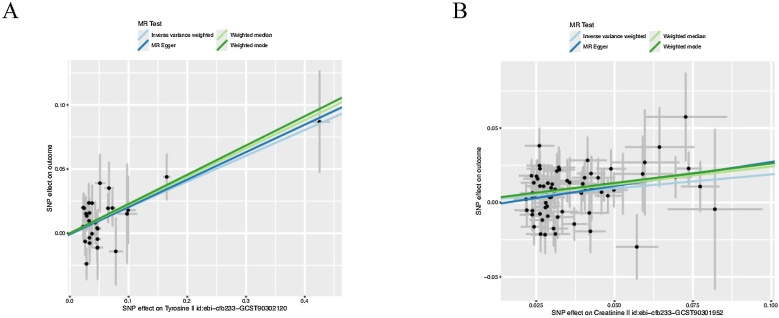
Two-sample Mendelian randomization analysis of Tyrosine and Creatinine (exposure traits) on CRS (outcome).

**Fig. 4:**
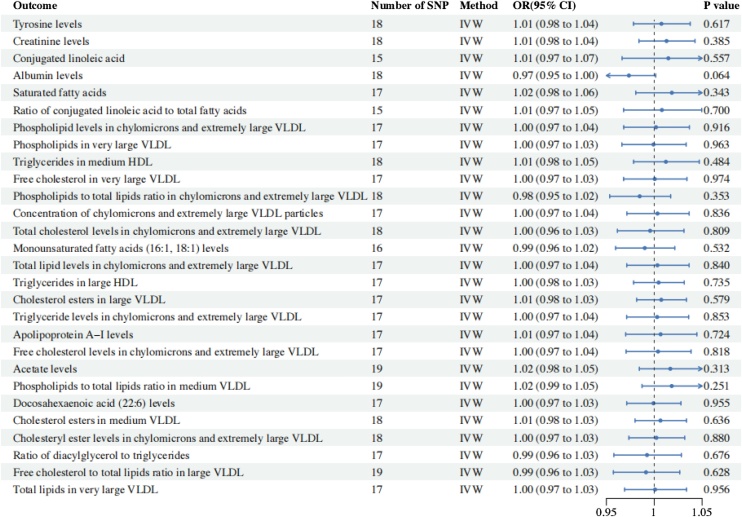
Reverse Mendelian randomization estimates of CRS and Circulating metabolites.

We also found some circulating metabolites with suggestive associations with CRS. Acetate (OR = 1.263; 95% CI 1.021–1.562; *p* = 3.15E-02), Concentration of medium VLDL particles (OR = 1.093; 95% CI 1.01–1.19; *p* = 0.036) were positively with CRS incidence. Alternatively, several metabolites suggest a protective factor for CRS, including Ratio of conjugated linoleic acid to total fatty acids (OR = 0.809; 95% CI 0.708‒0.923; *p* = 1.73E-03), Albumin (OR = 0.787; 95% CI: 0.670‒0.926; *p* = 3.76E-03), Ratio of saturated fatty acids to total fatty acids (OR = 0.901; 95% CI: 0.839‒0.967; *p* = 3.85E-03), Conjugated linoleic acid (OR = 0.664; 95% CI: 0.491‒0.898; *p* = 7.85E-03), Phospholipids in chylomicrons and extremely large VLDL (OR = 0.915; 95% CI: 0.856‒0.977; *p* = 8.23E-03), Phospholipids in very large VLDL (OR = 0.912; 95% CI: 0.850‒0.979; *p* = 1.08E-02), Triglycerides to total lipids ratio in medium HDL (OR = 0.929; 95% CI: 0.877‒0.985; *p* = 1.30E-02), Free cholesterol to total lipids ratio in very large VLDL (OR = 0.914; 95% CI: 0.850‒0.983; *p* = 1.48E-02), Concentration of chylomicrons and extremely large VLDL particles (OR = 0.879; 95% CI: 0.792‒0.975; *p* = 1.50E-02), Phospholipids to total lipids ratio in chylomicrons and extremely large VLDL (OR = 0.920; 95% CI: 0.860‒0.985; *p* = 1.69E-02), Cholesterol esters to total lipids ratio in chylomicrons and extremely large VLDL (OR = 0.915; 95% CI: 0.851‒0.985; *p* = 1.80E-02), Ratio of monounsaturated fatty acids to total fatty acids (OR = 0.927; 95% CI: 0.869‒0.989; *p* = 2.18E-02), Total lipids in chylomicrons and extremely large VLDL (OR = 0.920; 95% CI: 0.856‒0.989; *p* = 2.34E-02), Triglycerides to total lipids ratio in large HDL (OR = 0.926; 95% CI: 0.867‒0.990; *p* = 2.47E-02), Total cholesterol to total lipids ratio in large VLDL (OR = 0.931; 95% CI: 0.873−0.992; *p* = 2.81E-02), Triglycerides to total lipids ratio in chylomicrons and extremely large VLDL (OR = 0.921; 95% CI: 0.856‒0.991; *p* = 2.85E-02), Apolipoprotein A–I (OR = 0.915; 95% CI: 0.845‒0.991; *p* = 2.89E-02), Free cholesterol to total lipids ratio in chylomicrons and extremely large VLDL (OR = 0.921; 95% CI: 0.855‒0.992; *p* = 3.05E-02), Ratio of 22:6 docosahexaenoic acid to total fatty acids (OR = 0.919; 95% CI: 0.848‒0.995; *p* = 3.72E-02), Total cholesterol to total lipids ratio in medium VLDL (OR = 0.933; 95% CI: 0.874‒0.996; *p* = 3.79E-02), Total cholesterol to total lipids ratio in chylomicrons and extremely large VLDL (OR = 0.928; 95% CI: 0.864‒0.996; *p* = 3.84E-02), Diacylglycerol (OR = 0.804; 95% CI: 0.654−0.989; *p* = 3.87E-02),Free cholesterol in large VLDL (OR = 0.901; 95% CI: 0.815‒0.995; *p* = 3.96E-02), Total lipids in very large VLDL (OR = 0.929; 95% CI: 0.863–1.000; *p* = 4.89E-02) (Fig. 2). The scatter plot of the two metabolites that are in line with the correction, Tyrosine and Creatinine, shows that there is no horizontal hysteresis in the CRS, and the relationship is positively correlated (Fig. 3). After that, we used reverse MR Analysis. But we did not find any significant reverse MR positive results, this suggests that changes in CRS levels don’t influence circulating metabolites levels, reinforcing the conclusion of a one-way causal relationship (Fig. 4). Disruption of circulating metabolites indicates systemic imbalances that may impact immune cells, contributing to epithelial tissue damage in CRS.^7^ This immune dysregulation may exacerbate CRS by promoting inflammation and impairing the normal repair processes of the epithelial barrier.^8^ Understanding the interplay between circulating metabolites and immune responses is crucial for elucidating the pathophysiology of CRS and may provide insights into novel therapeutic strategies.

### Sensitivity analysis

**Fig. 5:**
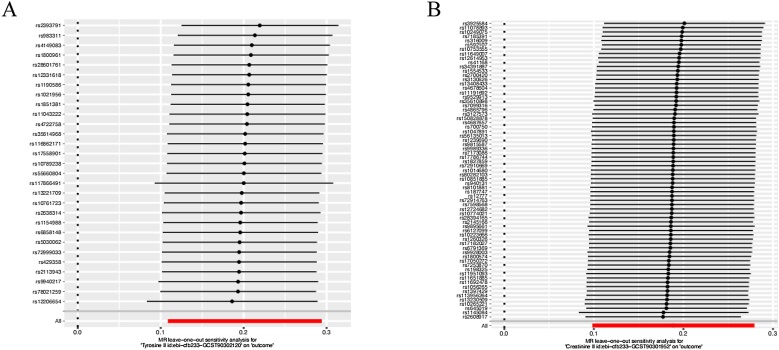
MR leave-one-out sensitivity analysis for tyrosine and creatinine.

We conduct sensitivity analysis to determine the reliability of IVW results. MR-Egger method is used to determine that no horizontal pleiotropy exists between CRS and 28 circulating metabolites (*p* > 0.05) (Table 1). In addition, we used a leave-one-out analyses to analyze two key statistically significant circulating metabolites separately and found that these estimates were not driven by any single SNP, indicating that the estimates were not compromised (Fig. 5).

**Table 1:** The result of sensitivity analysis.

Exposure	Heterogeneity *p-*value	MR-Egger intercept	Pleiotropy *p*-value
Tyrosine levels	0.511050256	−0.000899871	0.831720281
Creatinine levels	0.205808801	−0.005060188	0.310760309
Conjugated linoleic acid	0.610145221	0.005196209	0.641802105
Albumin levels	0.03714632	−0.003082091	0.672199006
Saturated fatty acids	0.16102408	−0.009196833	0.019655961
Ratio of conjugated linoleic acid to total fatty acids	0.825374267	−0.006457805	0.876533692
Phospholipid levels in chylomicrons and extremely large VLDL	0.558022329	0.002176479	0.525981411
Phospholipids in very large VLDL	0.186150021	0.000995859	0.778366825
Triglycerides in medium HDL	0.821989673	−0.001629403	0.620421493
Free cholesterol in very large VLDL	0.170356639	0.003483329	0.350292699
Phospholipids to total lipids ratio in chylomicrons and extremely large VLDL	0.783823704	0.004655275	0.489855341
Concentration of chylomicrons and extremely large VLDL particles	0.34032034	0.002310062	0.526144224
Total cholesterol levels in chylomicrons and extremely large VLDL	0.070962445	0.002656131	0.496507601
Monounsaturated fatty acids (16:1, 18:1) levels	0.139052831	−0.004715905	0.178777234
Total lipid levels in chylomicrons and extremely large VLDL	0.191664421	0.003058033	0.42419648
Triglycerides in large HDL	0.261975135	−0.003707638	0.296735541
Cholesterol esters in large VLDL	0.072459608	−0.000212054	0.950077579
Triglyceride levels in chylomicrons and extremely large VLDL	0.196913473	0.003321069	0.409502353
Apolipoprotein A–I levels	0.000323466	−0.001059741	0.78471573
Free cholesterol levels in chylomicrons and extremely large VLDL	0.16841085	0.004274006	0.25743338
Acetate levels	0.632524882	−0.003431578	0.776100443
Phospholipids to total lipids ratio in medium VLDL	0.082832621	0.007224283	0.109423675
Docosahexaenoic acid (22:6) levels	0.250470675	−0.008403062	0.051029249
Cholesterol esters in medium VLDL	0.026818314	0.000137442	0.968058597
Cholesteryl ester levels in chylomicrons and extremely large VLDL	0.092374996	0.001997252	0.591358778
Ratio of diacylglycerol to triglycerides	0.302264401	0.006633795	0.690675203
Free cholesterol to total lipids ratio in large VLDL	0.337304556	−0.006919603	0.341412558
Total lipids in very large VLDL	0.055082262	0.003946978	0.258040852
Tyrosine levels	0.511050256	−0.000899871	0.831720281

## Discussion

Our study used MR analysis to identify 28 circulating metabolites associated with CRS. This is the first discovery of metabolic cycles related to CRS. Among them, 2 were strongly associated with CRS, 4 were risk factors leading to CRS aggravation, and 24 circulating metabolites had protective effects on CRS. Compared to traditional observational studies, MR effectively reduces the risk of confounding variables and reverse causation.

We identified a significant correlation between tyrosine and creatinine levels and CRS risk, revealing previously overlooked associations. This finding aligns with studies suggesting these metabolites may contribute to inflammation, as seen in conditions like multiple sclerosis, where excess tyrosine oxidation leads to nitro tyrosine accumulation.^31^, ^32^ Meta-tyrosine and ortho-tyrosine in tyrosine can be markers of oxidative stress, and when their content increases, it represents the enhancement of oxidative stress.^33^ The continuous onset of CRSwNP and the occurrence of nasal polyps are likely to be related to oxidative stress.^34^ Therefore, there is some theoretical support that tyrosine may be involved in the pathogenesis of CRS in our MR Analysis results. Research has shown that arachidonic acid, a type of polyunsaturated fatty acid, can regulate chemokines and alleviate inflammation in CRS.^35^ However, in our study, we found no evidence of the effect of circulating polyunsaturated fatty acids on CRS.

Creatinine, a product of creatine phosphate metabolism in muscles, serves as a clinical marker for renal function, thyroid function, and muscle injury.^36^ In a mouse model of induced sepsis, activation of the IL-2 inducible T-cell Kinase (ITK) signaling pathway elevated creatinine levels.^37^ ITK inhibition is a potential treatment for T-cell-mediated inflammatory diseases such as rheumatoid arthritis and can restore the balance between Th17 and regulatory T (Treg) cells.^14^ Additionally, deactivation of the ITK gene has shown benefits in inflammatory disease models like asthma and colitis by reducing disease severity.^38^, ^39^ In CRSwNP, CD4+ T-cell recruitment in the nasal mucosa and Treg cell-mediated regulation of the Th1/Th2 balance are crucial in polyp formation.^40^, ^41^ Increased creatinine may alter T-cell function in CRS via ITK regulation, while acetate enhances CD4+ T-cell differentiation through increased GAPDH activity.^42^, ^43^, ^44^ Elevated blood acetate levels may stimulate cytokine production following CD4+ T-cell differentiation, although the precise immunomodulatory effects remain elucidated.

Current CRS treatment mainly focuses on symptom management. Our findings indicate that risk metabolites or protective metabolites as biomarkers for risk stratification, patients can be classified into mild, moderate, and severe categories. Changes in the levels of these metabolites may serve as indicators of disease remission or exacerbation. Finally, personalized treatment strategies can be implemented through varying degrees of intervention, such as dietary modifications or pharmacological approaches, to adjust the incidence or severity of CRS. We identified 24 circulating metabolites that are negatively associated with CRS, suggesting a potential protective role. CLA has been shown to reduce Th2 type immune responses, for example incorporating CLA into the diet of rats results in a reduction of allergy-related Ig-E levels.^15^ In vitro, CLA exposure inhibited DC migration and Th cell activation, reducing IL-17 secretion from T-cells.^1^, ^45^, ^46^ Cluster analysis of inferior turbinate tissues from CRS patients revealed that 4 biomarkers, including albumin, exhibited low concentrations.^47^ ECRSwNP has shown higher albumin levels in nasal polyps compared to non-eosinophilic CRSwNP. Apolipoprotein A–I (ApoA–I), a multifunctional component of HDL, has anti-inflammatory effects in immune-mediated diseases. In systemic lupus erythematosus-like mouse models, ApoA–I deficiency reduces plasma HDL levels, activating dendritic cell inflammation and enhancing T-cell cytokine secretion.^48^, ^49^ Diacylglycerol (DAG) acts as second messenger in T-lymphocyte activation.^50^ DGKζ deletion increases DAG levels, reducing Th2 cell cytokines and eosinophils in bronchoalveolar lavage fluid, inhibiting mast cell degranulation, and decreasing cytokine release from macrophages.^51^, ^52^ While DAG balances Th1 and Th2 cells to alleviate immune responses.

Our study has several limitations. First, because the GWAS data used in this analysis were derived from subjects of European ancestry, the generalizability of our findings to other populations is limited. Therefore, there is a critical need for further validation studies in diverse populations. Second, due to the limitations of the database, we were unable to discern which specific subtype of CRS was associated with the circulating metabolites. Third, although our MR analysis has some preliminary evidence to support it, further basic research is still needed to validate the findings.

Our study introduces significant novelty by identifying previously unknown circulating metabolite associations with CRS, expanding the understanding of its pathogenesis and suggesting that metabolic dysregulation may be critical to the disease's development and progression. This insight not only enhances our understanding of disease mechanisms but also opens new research avenues for targeting metabolic pathways therapeutically. Future research should include longitudinal studies to explore changes in circulating metabolites in CRS and their links to symptoms and prognosis. Further investigation is needed into metabolites, like CLA in Th2-type immune responses. Additionally, RCTs should assess the effectiveness of dietary interventions targeting these metabolites to improve CRS symptoms or prognosis.

## Conclusion

In our MR study, we identified 28 circulating metabolites associated with CRS, among 4 were positively correlated with CRS, 24 were identified as protective factors against CRS. Tyrosine and creatinine emerged as the most robust pathogenic factors. It provides novel insights into the circulating metabolites involved in CRS pathogenesis. Importantly, our findings offer valuable insights for the personalized prevention and management of CRS, emphasizing the potential of metabolic analysis to provide more effective and tailored therapeutic approaches based on individual metabolic profiles. This foundation for personalized interventions opens new avenues for improving patient outcomes and underscores the urgent need for innovative strategies in CRS management.

## Ethics approval and consent to participate

This article utilizes data from studies involving human participants, where informed consent was obtained from all individuals in the original research. Our study primarily focuses on analyzing large-scale GWAS datasets instead of individual-level data, which eliminates the necessity for ethical approval in this case. Clinical trial number: not applicable. Clinical trial number: not applicable.

## Consent for publication

All authors have read the manuscript and have agreed to its publication.

## Funding

This study was supported by the 10.13039/501100001809National Natural Science Foundation of China (grant nos. 81460096, 81860182), Jiangxi Nutrition and Health Management Medical Research Institute Cultivation Project (2022-PYXM-05) and Central Funds Guiding the Local Science and Technology Development (20221ZDG020066).

## Availability of data and material

Data contained in the manuscript are provided within the article. Public data can be found here: (https://www.finngen.fi/en/access_result).

## Declaration of competing interest

The authors declare no conflicts of interest.
